# Fast, easy, cheap, robust and safe method of analysis of Sudan dyes in chilli pepper powder

**DOI:** 10.1016/j.heliyon.2020.e05243

**Published:** 2020-10-13

**Authors:** Joseph Kweku Adjei, Vigil Ahormegah, Alex Kissi Boateng, Harry Kwaku Megbenu, Samuel Owusu

**Affiliations:** a Department of Chemistry, University of Cape Coast, Cape Coast, Ghana; b Department of Laboratory Technology, University of Cape Coast, Cape Coast, Ghana

**Keywords:** Food analysis, Food safety, Analytical chemistry, Sudan dyes, Chilli pepper powder, Modified QuEChERs, UFLC-UV

## Abstract

Illicit use of Sudan dyes, a group of harmful and carcinogenic azo dyes, in the food industry has taken a surge in various parts of the world, especially in Africa. Their use in food as additives pose a dire health risk to consumers and have been banned by various food regulatory bodies worldwide. To help increase surveillance, various methods have been proposed for their analysis in literature. This study also sought to experiment and propose an alternative method for quick, easy, cheap, robust and ecologically safe analysis of Sudan dyes in chilli pepper powder and similar matrices. The optimized method used a 6.0 mL mixture of acetone:acetonitrile (1:5 v/v) solvent in a modified QuEChERs method for extraction of Sudan dyes I-IV. The simultaneous analysis of the dyes were achieved on Shimadzu prominence UFLC 20AD coupled with SPD 20AX UV detector operated at dual wavelength of 500 and 480 nm. A total of twenty four (24) chilli pepper powder samples from eight different vendors on the Ghana market were analysed using the optimized method. Quantitation of analytes were done using the external standard calibration method with determination coefficient, R^2^ > 0.9999. The limit of detection (LOD) and limit of quantitation (LOQ) of the method were 0.02–0.04 mg/kg and 0.05–0.13 mg/kg respectively. A good recovery range between 85.3 – 121.2% were obtained for a spike level of 1.0 mg/kg in real samples. ANOVA analysis at 95% CL showed statistically no significant difference (p > 0.05) in the recoveries between samples and also between the individual compounds. The method experimented and proposed in this study is fast, easy, cheap, robust and ecologically safe, presenting an alternative method for routine analysis for increased rate of surveillance against the illicit use of Sudan dyes as food additives.

## 1 Introduction

Sudan dyes are synthetic organic dyes (azo dyes) used industrially to colour textiles, plastics, shoe leather and other synthetic products such as floor polish. They are cheap, highly lipophilic and have attractive intense red colours, hence have illegally been used in some food industry as food additives to enhance the colour of foods such as palm oil, spices, powder Chilli pepper and sauces as well as some meat products (Limin et al., 2007; Chailapakula et al., 2008; Qi et al., 2011; Genualdi et al., 2016; Zhang et al., 2016; Yang et al., 2019). The most commonly used and investigated compound in foods, especially Chilli pepper, among these azo dyes are Sudan I, II, III, and IV (Zheng et al., 2007; Ávila et al., 2011; Qi et al., 2011). The presence of Sudan dyes in food presents a serious public health hazard and also has dire economic implication (Nisa et al., 2016). Because of the several detrimental health effects associated with Sudan dyes as adulterants in foods on consumers, various international and independent regulatory bodies have placed a ban or stringent limits on the use of these dyes as food additives (2003/460/EC; 2004/92/EC). The IARC in 1987 (IARC, 1987), classified Sudan I – IV as class 3 human carcinogen. The European Food Safety Authority (EFSA) in its 2005 review of toxicological potency of several illegal colorants used as food additives concluded that Sudan I especially and for that matter II, III and IV because of their structural similarity are both genotoxic and carcinogenic, which was also assented by IARC in its 2010 monograph (IARC, 2010). McCann et al. (2007) reported that the consumption of Sudan dyes in food is associated with hyperactive behaviour and loss of concentration in children. Johnson et al. (2010) and An et al. (2007) also reported the genotoxic effect of Sudan I in their studies. The European Union (EU) in its early reports in 2003/2004 sanctioned the mandatory testing of Sudan I, II, III and IV in all hot powdered Chilli pepper and their products (2003/460/EC; 2004/92/EC) because of their associated health effect on consumers. Additionally, the EU in 2006, considering the wide spread report of Sudan dyes in foodstuff set the maximum permissible limits of all Sudan dyes to be 0.5 mg/kg (2006/33/EC).

Unfortunately, to date, the menace of using these carcinogenic azo dyes as food additives in the food industry has not stopped. For instance, in late 2006 the presence of Sudan IV dye was reported in some duck and hen eggs in Mainland China (Centre for food safety CFS, 2006). Amoako-Mensah (2017) reported elevated levels of Sudan IV dye in palm oils on the Ghanaian market.

Because of prevalence illegal use of Sudan dyes in foods, and to enhance surveillance, several analytical methods have been proposed in literature (Ertas et al., 2007; Di Anibal et al., 2009; Ávila et al., 2011; Sun et al., 2011; Qi et al., 2011; Li et al., 2014; Yang et al., 2019). Most of the methods proposed to help in this regard took into consideration one or two of the following merits, i.e. rapidity, robustness, and cost, while compromising on one or more of these merits. For instance rapid and efficient sample treatment and analytes extraction is a prevailing challenge in real sample analysis and the cost involved cannot be overemphasised. This study therefore sought to experiment and propose a method that factors all these merit mentioned earlier to help with testing and increase surveillance. That is a method which is rapid, easy, cheap, and robust as well as environmentally safe considering the amount and nature of reagents and solvent used. Though this method may be applicable to other food matrices, the study basically dwelled more on rapid and efficient sample extraction and ultrafast liquid chromatography-ultraviolet (UFLC-UV) analysis of four Sudan dyes, namely Sudan I, II, III and IV in Chilli pepper samples.

## 2 Material and methods

### 2.1 Reagents and standards

Single component Sudan dye standards (purity >96.0%) for Sudan I, II, III, and IV (Sigma Aldrich). GC grade acetone (purity >99.0%), and LC grade acetonitrile (purity >99.9%) were purchased from Merck KGaA, Darmstadt, Germany. The glacial acetic acid (purity >99.7%) was from Daejung Chemicals, Siheung, Korea. MilliQ ultrapure deionized water (18.2 MΩ) filtered through MilliQ polisher (DI water), AOAC Method 2007.01 roQ QuEChERS extraction salts (6.0 g MgSO_4_ + 1.5 g NaOAc) were purchase from Phenomenex Inc.

### 2.2 Samples

The chilli pepper powder samples were randomly purchased from two major regions in Ghana, namely Ashanti and Greater Accra region. A total of twenty four (24) different samples were purchased from eight vendors. Spiked and unspiked samples were prepared for each sample, and extracted for the analysis.

#### 2.2.1 Sample preparation

The samples were extracted using three (3) different types of solvent systems, i.e. 6.0 mL acetonitrile only, 1.0 mL acetone and 5 mL acetonitrile mixture [17:83% (v/v)] and 6.0 mL acetone: acetonitrile [50:50%(v/v)]. This was done to optimize the extraction process with the right solvent combination for efficient and reproducible extraction.

In all extractions, 2.0 g of powdered chilli pepper samples were taken. Spiked samples at 1.0 mg/kg spike levels were prepared by the addition of a mix Sudan dye standards. A volume of 2.0 mL DI water was added to the sample in a centrifuge tube. The sample was shaken for 30 s and 6.0 mL of the extraction solvent was added followed by the addition of 2.0 g QuEChERS extraction salt mixture. The resulting mixtures in the tubes were manually shaken for 1.0 min and finally centrifuged at 4000 rpm for 5 min. Prior to UFLC-UV analysis, the supernatants were filtered through a 0.45 micron syringe filter and 1.5 mL was taken for the analysis. All samples both spiked and unspiked were extracted and analysed in replicates (n = 3). A reagent blanks were also extracted in similar manner and analysed.

### 2.3 Working standard preparation

A 1000 mg/kg of a mix standard was prepared by dissolving 0.1 g of each Sudan dye standard in acetonitrile and made up to the mark in a 100 mL volumetric flask. An amount of the stock solutions was taken and diluted with acetonitrile to prepare a working standards of concentrations of 0.1, 0.5, 1.0, 2.0, 5.0, and 10.0 mg/kg. Continuous calibration verification standards of 10.0 mg/kg were also prepared daily. Also a 10.0 mg/kg standard for system suitability was prepared.

### 2.4 Ultrafast liquid chromatography coupled with ultra-violet detector (UFLC-UV) analysis

The analysis was carried out using Shimadzu Prominence UFLC 20AD coupled with UV detector SPD 20AX.

#### 2.4.1 Analytical conditions

The solvent systems used for elution were:

Mobile phase A: 0.1% (v/v) acetic acid in DI water.

Mobile phase B: Acetonitrile.

Elution Solvent mixture: A:B = 10:90% (V/V).

The LC system was operated in isocratic mode with a flow rate 1.5 mL/min. The column used was 150 mm × 4.6 mm (i.d.) Phoenix C-18 column and it was maintained at a temperature of 40 °C in an oven.

The UV-Vis detector SPD-20AX was operated in dual wavelength mode, i.e. 500 nm (channel 1) and 480 nm (channel 2). The flow cell temperature was maintained at 40 °C.

The injection volume for standards and samples was 5.0 μL and the total run and detection time was 10 min.

#### 2.4.2 Data acquisition

All the UFLC-UV analytical data were acquired using Labsolution version 3.0 software. Data were obtained by comparing retention time and area of each compound in the standards with that of each real sample. The external calibration method was used in acquiring concentration levels.

#### 2.4.3 Quality control

System suitability was conducted for each batch using FDA Criteria. Solvent A, was freshly prepared prior to each daily batch of analysis. Spiked and unspiked samples were analysed in replicates. Reagent blank was prepared and analysed in a similar manner to the samples used. Initial Calibration verification (ICV) standards of 0.4 and 2.0 mg/kg concentrations were ran to initially validate the calibration curve. Continuing calibration verification standard at 10.0 mg/kg concentration were also ran in triplicates (n = 3) after each ten (10) sample analysis to validate the calibration curve.

### 2.5 Statistical data analysis

Analysis of variance (ANOVA) and other statistics on the data were conducted with excel toolpak. The Shimadzu labsolution browser was also employed in the calculations of LOD, LOQ and the statistics of the other analytical merits.

## 3 Results and discussion

### 3.1 Quality control results/analytical figure of merits

**Figure 1:**
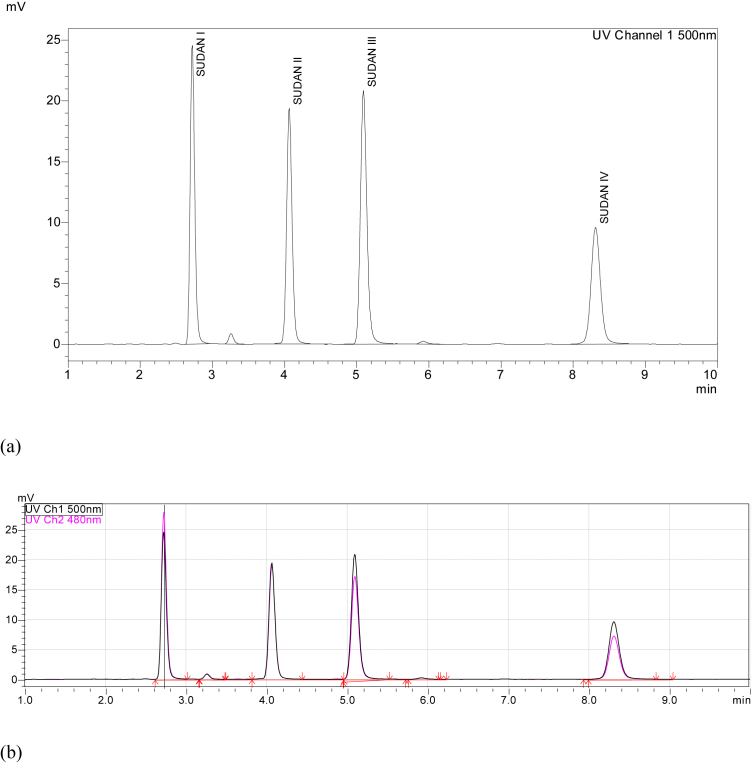
A chromatogram of 10.0 mg/kg Sudan I-IV dyes mixture (a) at only 500 nm and (b) dual wavelength of 500 nm and 480 nm.

Table 1 showed a pass for all the system suitability parameters, which is an indication of a good instrumental analysis practice, which ensured selectivity and robustness of the instrumental method for separating and quantitation of the analytes from other matrices. Well resolved peaks were achieved for this study as shown in Figure 1. The optimized method had Sudan I and II absorbing optimally at an absorbance of 480 nm in the visible region [Figure 1(b)].

**Table 1:** FDA System suitability criteria and results of this study (n > 10).

Parameter	Recommendation	This study
Retention factor, *k*	*k* > 2	k > 2
Injection repeatability	%RSD ≤1% for n ≥ 5	RSDs <0.6%,
Resolution, R_s_	R_s_ >2	>4.0
Tailing factor, TF	TF ≤ 2	≤1.2
Column plate number, N	N > 2000	N > 10000

**Figure 2:**
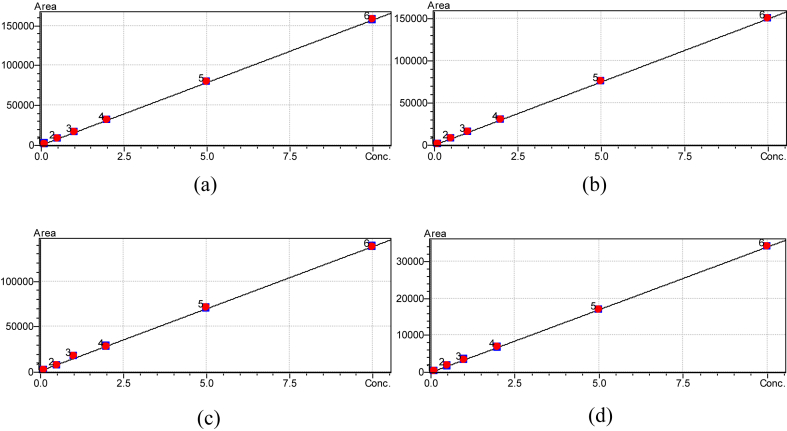
Six point calibration curves for Sudan dyes I(a), II(b), III(c) and IV(d).

The six point calibration curves were linear between the range of 0.10–10.00 mg/kg and the coefficients of determination, R^2^ were greater than 0.9999 (Figure 2; Table 2). The RF %RSDs for the calibration curves were less than 5% (Table 2). These results are indications of a very good linearity in analytical response of the method employed. The detection limits and the quantitative limits were in the ranges 0.02–0.04 mg/kg and 0.05–0.13 mg/kg respectively (Table 2). These limits indicated that the method employed herein had improved sensitivity when compared to EU set quantitative limits of 0.5–1.0 mg/kg. The improved sensitivity was also comparable to the 16.6–36.9 μg/kg detection limit reported by the National Standards of the People's Republic of China (GB/T 19681-2005) in similar work. The analytical sensitivity also recorded a significant improvement when compared to result from Cornet et al. (2006) where DL of 0.20–0.30 mg/kg were obtained in similar works and also to that of Ávila et al. (2011), Li et al. (2015) and Yang et al. (2019), where further higher values were reported as LODs and LOQs.

**Table 2:** Mean values of analytical quality control parameters (merits) for the analysis.

Compound name	Retention time	Equation for calibration curves, (10^3^)	R^2^	RF %RSD	Detection limit (DL), mg/kg	Quantitative limit (QL)
Sudan I	2.72	y = 15.688x + 0.325	0.99999	3.356	0.02	0.05
Sudan II	4.06	y = 14.968x + 0.138	0.99999	1.841	0.02	0.06
Sudan III	5.10	y = 17.701x – 0.318	0.99998	3.836	0.02	0.07
Sudan IV	8.43	y = 3.403x – 0.324	0.99999	2.466	0.04	0.13

#### 3.1.1 Other figures of merits

The ICVs recorded mean values ranging from 0.399 ± 0.02 to 0.40 ± 0.02 mg/kg and 1.99 ± 0.002 to 2.02 ± 0.039 mg/kg, indicating a recovery of about 99.5–100.9% and 99.8–100.8% respectively for 0.4 and 4.0 mg/kg of standards analysed (Table 3). The CCV standards analysed for inter day check of the calibration curves accuracy, also had recovery values range between 100.1 – 103.0% (Table 3). The blue regions of the calibration curves’ plot dots also indicated the good precisions (RSD <0.5) of the instrumental analysis of the four Sudan dyes (Figure 2).

**Table 3:** A table showing mean ICVs recoveries (n = 3) and mean CCVs recoveries (n = 10).

Compound name	Mean ICV at 0.4 mg/kg (% recovery)	Mean ICV at 2.0 mg/kg (% recovery)	Mean CCV(mg/kg), Recoveries, (%)
Sudan I	0.40 ± 0.02 (100.9)	1.99 ± 0.002 (99.8)	10.09 ± 0.014 (100.9)
Sudan II	0.40 ± 0.01 (100.1)	2.02 ± 0.039 (100.8)	10.01 ± 0.011 (100.1)
Sudan III	0.40 ± 0.02 (99.5)	2.00 ± 0.040 (100.0)	10.25 ± 0.16 (102.5)
Sudan IV	0.40 ± 0.03 (99.9)	2.01 ± 0.004 (100.4)	10. 29 ± 0.20 (103.0)

These results indicated that a good accuracy, repeatability and reproducibility for the instrumental method was achieved in the analysis. This also indicated that the method is rugged and very reliable. The results are comparable to that reported by Qi et al. (2011) where a mean recovery of 93.2–103.0 % were obtained.

### 3.2 Extraction optimisation

Extraction optimization conducted to obtain the right solvent system for optimum results gave promising results for all the solvents used.

The results (Table 4) showed that a combination of acetonitrile and acetone make a better solvent system for efficient extraction of Sudan I-IV dyes in powdered chilli pepper than using acetonitrile alone. The effect was very significant especially in the extraction of the more lipophilic dyes, Sudan III and IV, where 50:50% (v/v) acetone: acetonitrile solvent system were found to have greater recovery than the other solvent system (Table 4), which was followed closely by 1:5 mL (17:83% v/v). This suggested that the higher the percent component of acetone, the better the recovery of Sudan III and IV. Two-way ANOVA conducted at 95% confidence level (CL), suggested that there were statistically significant difference (p = 0.015) between the mean recoveries of the individual Sudan dyes per each extraction solvent, but no significant difference (p = 0.06) were observed between the solvent systems for the mean total percent extraction recovery of the Sudan dyes. On the whole, the total mean recovery of 1:5 mL acetone:acetonitrile solvent system was found to be better with a mean recovery of 98.3% for the analytes than the others (Table 4). It was therefore chosen as the best solvent system for the analysis of the rest of the real chilli pepper samples.

**Table 4:** Initial percent mean extraction recovery of each solvent system (n = 5).

Compound name	Mean (100% ACN, 6 mL), %	Mean [1:5 mL (17:83% v/v) acetone:ACN, 6 mL], %	[3:3 mL (50:50% v/v) acetone: ACN, 6 mL], %
Sudan I	100.9	107.3	106.9
Sudan II	95.0	104.9	93.3
Sudan III	81.3	96.6	92.4
Sudan IV	69.7	84.5	93.4
Mean	86.7	98.3	96.5

### 3.3 Analysis of other real samples and extraction recoveries

**Figure 3:**
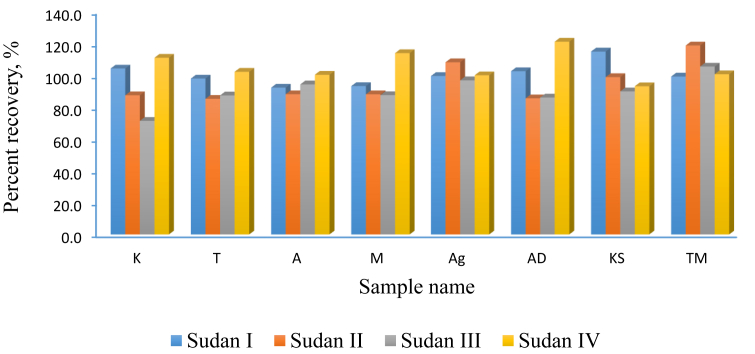
The mean percent recovery (n = 3) of Sudan dyes from real chilli pepper samples.

The methods were employed in the further analysis of twenty four (24) real chilli pepper samples from eight different major vendors on the Ghanaian market. The mean percent recoveries (n = 3) of the spiked samples ranged between 85.3 – 121.2% (Figure 2). Sudan IV recorded relatively higher recoveries (>100%) than Sudan I, II and III. This indicated that the method is efficient in the analysis of Sudan IV, which is illicitly widely used as food colorant due to it intense red colour. The results (Figure 3) in this study were comparable to the results obtained by Qi et al. (2011) in similar work. Similar recoveries ranging between 85 - 101 % were also reported by Baggiani et al. (2009) in similar study where molecularly imprinted SPE was employed. Zheng et al. (2011) reported a recovery between 85.6 -119.7% in similar study where SPE extraction coupled with direct time-of-flight mass spectrometry were employed. Better recoveries were achieved in this study than that reported by Genualdi et al. (2016), where recoveries as low as 66.0% were obtained in some cases (Table 5). It is worth noting that this current study recorded higher recoveries in some cases especially for Sudan IV than reported by earlier studies (Table 5). Moreover, the method in this current study was quicker than similar methods reported in literature, employing only a maximum total time of 20 min for both extraction and instrumental analysis. It thus help save time for routine analysis of samples thereby increase productivity and surveillance rate in the fight against the illicit use of Sudan dyes in the food and drug industries.

**Table 5:** A comparison of the proposed method to similar methods in Literature.

Analytical Method	LOD (mg/kg)	RSD %	Recovery, %	Reference
Modified QuEChERs Extraction-UFLC-UV	0.020–0.04	1.8–3.4	85.3–121.2	This study
Liquid-liquid extraction-LC-DAD-MS/MS	3.10–4.60	Not reported	66.0–104	Genualdi et al. (2016)
supercritical fluid extraction–capillary liquid chromatography (SFE-CLC)	0.023–0.042	6.8–11.6	91.0–109.0	Ávila et al. (2011)
HPLC-DAD	0.004–0.006	0.3–4.4	93.2–103.0	Qi et al. (2011)
Solid Phase Extraction-TOF-MS	0.001–0.004	<11.6	85.6–119.7	Zheng et al. (2011)
Direct analysis in a real time-mass spectrometry (DART-MS)	~0.5	<26.0	88.0–116.0	Li et al. (2015)
Sonication-Centrifugation-HRMS	0.5–1.0	<20.0	Not stated	Sciuto et al. (2017)
Ultra-high-performance Supercritical fluid chromatography (UHPSFC)-PDA	0.10–0.30	<8.0	82.6–108.3	Yang et al. (2019)
Centrifugation-UPLC-MS/MS	0.003–0.03	2.24–12.2	80.7–104.4	Wang et al. (2013)

It is also worth noting that the use of a maximum total organic solvent volume of 6.0 mL for extraction, may pose a minimal risk to laboratory personnel and the environment. The method may thus be said to be ecologically safe for use in all settings.

Two-way ANOVA conducted at the 95% CL showed no statistical significant difference (p > 0.05) between samples with respect to the total Sudan dyes recovery and also between the recoveries of each Sudan dye analytes in each sample. These suggested, the method employed in this study had good reproducibility and repeatability and may produce consistent recoveries for Sudan dyes in powdered chilli pepper samples and similar matrices.

## 4 Conclusion

This study employed a modified QuEChERs extraction protocol combined with UFLC-UV simultaneous analysis of the four Sudan dyes. The method, both extraction and instrumental analysis, was quick using a maximum total time of twenty minutes (20 min). The method was also robust and cheap when compared with other traditional and recent methods published in literature. The use of only 6.0 mL of organic solvent for extraction, poses a minimal risk to laboratory personnel and the environment. This method may help to increase productivity in the routine analysis of Sudan dyes in real samples, and help regulatory bodies in quick and accurate analysis of more samples per day to help with surveillance in the food industries to help curb the illicit use of these dyes.

## Declarations

### Author contribution statement

Joseph Kweku Adjei: Conceived and designed the experiments; Performed the experiments; Analyzed and interpreted the data; Contributed reagents, materials, analysis tools or data; Wrote the paper.

Vigil Ahormegah, Alex Kissi Boateng, Harry Kwaku Megbenu, Samuel Owusu: Performed the experiments; Contributed reagents, materials, analysis tools or data.

### Funding statement

This research did not receive any specific grant from funding agencies in the public, commercial, or not-for-profit sectors.

### Competing interest statement

The authors declare no conflict of interest.

### Additional information

No additional information is available for this paper.
